# Identification of Biochemical and Molecular Markers of Early Aging in Childhood Cancer Survivors

**DOI:** 10.3390/cancers13205214

**Published:** 2021-10-18

**Authors:** Silvia Ravera, Tiziana Vigliarolo, Silvia Bruno, Fabio Morandi, Danilo Marimpietri, Federica Sabatini, Monica Dagnino, Andrea Petretto, Martina Bartolucci, Monica Muraca, Eleonora Biasin, Riccardo Haupt, Marco Zecca, Franca Fagioli, Daniela Cilloni, Marina Podestà, Francesco Frassoni

**Affiliations:** 1 Stem Cell Laboratory and Cell Therapy Center, IRCCS Istituto Giannina Gaslini, 16147 Genoa, Italy; detiviglia@libero.it (T.V.); fabiomorandi@gaslini.org (F.M.); danilomarimpietri@gaslini.org (D.M.); federicasabatini@gaslini.org (F.S.); MonicaDagnino@gaslini.org (M.D.); marinapodesta@gaslini.org (M.P.); francescofrassoni@gaslini.org (F.F.); 2 Department of Experimental Medicine, University of Genoa, 16132 Genoa, Italy; silvia.bruno@unige.it; 3 Core Facilities-Clinical Proteomics and Metabolomics, IRCCS Istituto Giannina Gaslini, 16147 Genoa, Italy; andreapetretto@gaslini.org (A.P.); martinabartolucci@gaslini.org (M.B.); 4 Epidemiology and Biostatistics Unit and DOPO Clinic, IRCCS Istituto Giannina Gaslini, 16147 Genoa, Italy; monicamuraca@gaslini.org (M.M.); riccardohaupt@gaslini.org (R.H.); 5 Department of Pediatric Onco-Haematology, Regina Margherita Children’s Hospital, University of Turin, 10126 Turin, Italy; eleonora.biasin@unito.it (E.B.); franca.fagioli@unito.it (F.F.); 6 Pediatric Hematology Oncology, Fondazione IRCCS Policlinico San Matteo, 27100 Pavia, Italy; m.zecca@smatteo.pv.it; 7 Department of Clinical and Biological Sciences, School of Medicine, University of Turin, 10124 Turin, Italy; daniela.cilloni@unito.it; 8 Department of Mathematics (DIMA), University of Genoa, 16146 Genoa, Italy

**Keywords:** anticipated aging, childhood cancer survivors, energy metabolism, mitochondrial biogenesis, oxidative stress

## Abstract

**Simple Summary:**

Childhood cancer survivors (CCS) display a higher risk of developing second malignant tumors and chronic diseases compared with aged-matched controls because of chemo/radiotherapy. This early frailty seems associated with accelerated cell aging, a process correlated with altered mitochondrial energy production. Therefore, this work aims to shed light on the mechanisms involved in chemo/radiotherapy-induced early aging, morbidities, and the risk of developing second tumors in CCS through a biochemical and molecular approach. The identification of crucial mechanisms involved in the CCS chemo/radiotherapy-related pathological conditions will allow identifying therapeutic targets to develop appropriate risk-based care and interventions, minimize morbidities, and maximize the quality of life in the cancer survivor population.

**Abstract:**

Survival rates of childhood cancer patients have improved over the past four decades, although cancer treatments increase the risk of developing chronic diseases typical of aging. Thus, we aimed to identify molecular/metabolic cellular alterations responsible for early aging in childhood cancer survivors (CCS). Biochemical, proteomic, and molecular biology analyses were conducted on mononuclear cells (MNCs) isolated from peripheral blood of 196 CCS, the results being compared with those obtained on MNCs of 154 healthy subjects. CCS-MNCs showed inefficient oxidative phosphorylation associated with low energy status, and increased lipid peroxidation and lactate fermentation compared with age-matched normal controls. According to a mathematical model based on biochemical parameters, CCS-MNCs showed significantly higher metabolic ages than their real ages. The dysfunctional metabolism of CCS-MNCs is associated with lower expression levels of genes and proteins involved in mitochondrial biogenesis and metabolism regulation, such as CLUH, PGC1-alpha, and SIRT6 in CCS, not observed in the age-matched healthy or elderly subjects. In conclusion, our study identified some biochemical and molecular alterations possibly contributing to the pathophysiology of aging and metabolic deficiencies in CCS. These results identify new targets for pharmacological interventions to restore mitochondrial function, slowing down the aging-associated pathologies in CCS.

## 1. Introduction

Survival rates of childhood cancer patients have improved tremendously over the past four decades, since the 5-years survival rate is approaching 80% [1,2,3]. However, studies among childhood cancer survivors (CCS) clearly show the risks of long-term clinical complications related to chemotherapy, radiotherapy, or both [4,5,6]. It is likely that the damage to normal tissues from cancer therapies diminishes physiological reserves, thereby accelerating the processes associated with aging, and increasing several accumulated stressors that impair the ability to restore physiologic turn-over and cellular homeostasis [7]. In fact, several young adult CCS have reported frailty symptoms consistent with early aging [8,9,10], such as poor fitness, muscular weakness, poor exercise tolerance [11,12], and cognitive decline [13,14], appearing decades earlier than expected [4,15,16,17,18,19,20]. The two most prevalent severe health conditions in these young cancer survivors are cardiovascular disease [18,21] and malignant neoplasms [7], conditions associated with aging in the general population. However, the cellular/molecular basis of all described symptoms and signs [8,9,15] remains missing.

Aging is a process that involves several alterations, such as telomere shortening, systemic inflammation (e.g., C-reactive protein and IL-2 over-expression), deregulated autophagy, oxidative stress, metabolic dysfunction, and epigenetic modifications [22,23]. In this scenario, senescent cells accumulate dysfunctional mitochondria that increase the reactive oxygen species (ROS) production, determining a negative effect on cellular bioenergetics [24], and driving cell senescence [25]. The alteration of the oxidative phosphorylation (OxPhos) reduces the aerobic ATP production, inducing cells to switch to the anaerobic metabolism, probably to restore the cellular bioenergetics [26,27].

In this context, we generated a mathematical model able to predict the ages of individuals with a mean absolute error of approximately 9 years, based on the modifications of glucose catabolism observed in the mononuclear cells (MNCs) of the healthy population between 5 and 106 years of age [28]. Using this model, we investigated whether CCS are suffering from accelerated metabolic aging after cancer therapies. We focused on mitochondrial metabolism because their dysfunction determines the decrement of cellular energy availability and the imbalance between the generation of new mitochondria and their clearance, laying the foundation for aging.

## 2. Materials and Methods

### 2.1. Reagents

All chemicals were purchased from Sigma Aldrich (St. Louis, MO, USA), unless otherwise indicated. Ultrapure water (Milli-Q; Millipore, Billerica, MA, USA) was used throughout. All other reagents were of analytical grade.

### 2.2. Samples

All experiments were performed using MNCs isolated from peripheral blood (PB) with a standard gradient separation procedure within 24 h from collection. MNCs were chosen because they are considered an excellent model with which to evaluate the health status of an entire organism [29].

We studied 196 childhood cancer survivors after the end of treatment. This group of survivors had ages between 1 and 40 years old (y.o.): 103 with hematological malignancies (the commonest were acute lymphoblastic leukemia 60%, Hodgkin’s lymphoma 14%, and acute myeloid leukemia 11.5%) and 93 with solid tumors (neuroblastoma 59%, Wilms’ tumor 14.7%, rhabdomyosarcoma 10.3%, Ewing sarcoma 3.8%, others 12.2%). All patients were given prolonged chemotherapy and/or radiotherapy according to clinical trials for pediatric malignancies and were free of the disease at the time of the study. The median interval between completion of cancer treatment and blood sampling was 9 years (range: 2 months–25 years). Age-matched healthy donors with no cancer history (79, with ages between 1 and 40 y.o.) and older subjects (79, with ages between 41 and 106 y.o.) were tested as controls (Table 1).

This study was in accordance with the precepts established by the Helsinki Declaration and was approved by the Regional Ethics Committee of Liguria (number 096REG2014), and all participants provided their written informed consent. For the participants under the age of 18 years, we obtained informed consent from a parent and/or legal guardian. All the analyses were not performed blinded.

### 2.3. Evaluation of Oxygen Consumption, ATP Synthesis, P/O Ratio, ATP/AMP Ratio, and Lactate Dehydrogenase Activity

In these experiments, MNCs samples were analyzed within 24 h from collection.

The oxygen consumption rate (OCR) was measured with an amperometric O_2_ electrode in a closed chamber, magnetically stirred, at 37 °C (Unisense, DK). For each assay, 200,000 cells were used. Samples were suspended in a medium containing 137 mM NaCl, 5 mM KH_2_PO_4_, 5 mM KCl, 0.5 mM EDTA, 3 mM MgCl_2_, and 25 mM Tris–HCl, pH 7.4, and permeabilized with 0.03 mg/mL digitonin for 10 min. To stimulate the pathway composed of Complexes I, III, and IV, 5 mM pyruvate and 2.5 mM malate were added. To activate the pathway composed of Complexes II, III, and IV, 20 mM succinate was used [30].

ATP synthesis was measured with the highly sensitive luciferin/luciferase method. The assay was conducted at 37 °C, over 2 min, by measuring formed ATP from added ADP: 200,000 cells were added to the incubation medium (0.1 mL final volume) containing: 10 mM Tris-HCl pH 7.4, 50 mM KCl, 1 mM EGTA, 2 mM EDTA, 5 mM KH_2_PO_4_, 2 mM MgCl_2_, 0.6 mM ouabain, 0.040 mg/mL ampicillin, 0.2 mM p1p5-di(adenosine-5′) triphosphate, and the metabolic substrates—5 mM pyruvate plus 2.5 mM malate or 20 mM succinate. The cells were equilibrated for 10 min at 37 °C, then ATP synthesis was induced by the addition of 0.2 mM ADP. The ATP synthesis was measured using the luciferin/luciferase ATP bioluminescence assay kit CLSII (Roche, Basel, Switzerland), on a Luminometer (GloMax^®^ 20/20 Luminometer—Promega, Madison, WI, USA). ATP standard solutions (Roche, Basel, Switzerland) in the concentration range 10^−10^–10^−7^ M was used for calibration [28].

By dividing the nmol of ATP produced by the nmol of oxygen consumed in one minute, we obtained the P/O ratio, which indicates the efficiency of OxPhos metabolism. In coupled conditions, when the oxygen consumption is correctly associated with the ATP synthesis, this value is around 2.5, or 1.5 in the presence of pyruvate + malate or succinate [31]. Conversely, in the uncoupled status, this value decreases proportionally to the grade of the OxPhos inefficiency.

The ATP and AMP quantification was based on the enzyme coupling method [32]. For both assays, 20 µg of total protein was used. Briefly, ATP was assayed following NADP reduction, at 340 nm. The medium contained: 100 mM Tris-HCl pH 8.0, 1 mM NADP, 10 mM MgCl_2_, and 5 mM glucose in 1 mL final volume. Samples were analyzed spectrophotometrically before and after the addition of 4 µg of purified hexokinase plus glucose-6-phosphate dehydrogenase. AMP was assayed following the NADH oxidation at 340 nm. The medium contained: 100 mM Tris-HCl pH 8.0, 75 mM KCl, 5 mM MgCl_2_, 0.2 mM ATP, 0.5 mM phosphoenolpyruvate, 0.2 mM NADH, 10 IU adenylate kinase, 25 IU pyruvate kinase, and 15 IU of lactate dehydrogenase.

To assay the lactate fermentation flux, the activity of lactate dehydrogenase (LDH; EC 1.1.1.27) was measured at 25 °C on 20 µg of MNC homogenate. The reaction mixtures contained: 100 mM Tris-HCl pH 7.4, 0.2 mM NADH, and 5 mM pyruvate [33]. Enzymatic activity was expressed as mU/mg of total protein (nmol/min/mg of protein).

### 2.4. ROS Production and Lipid Peroxidation Evaluation

To evaluate the ROS level, freshly isolated MNCs were washed and re-suspended in PBS and stained for 10 min at 37 °C with 2′,7′-dichlorodihydrofluorescein diacetate (H2DCFDA) at a concentration of 5 µM (Thermo Fisher Scientific, Waltham, MA, USA). H2DCFDA is a non-fluorescent dye, which is cleaved inside cells to 2′,7′-dichlorofluorescein (H2DCF). In the presence of oxidants, H2DFC is converted in turn to the fluorescent compound DCF. Samples were measured on a FacsCalibur flow cytometer (Becton Dickinson, San José, CA, USA). The analysis was confined to viable cells only after gating based on forward- and side-scatter characteristics. Ten thousand cells per sample were analyzed [34].

Malondialdehyde (MDA) concentration was evaluated as a marker of lipid peroxidation, by the thiobarbituric acid reactive substances (TBARS) assay [35]. The TBARS solution contains: 15% trichloroacetic acid in 0.25 N HCl and 26 mM thiobarbituric acid. To evaluate the basal concentration of MDA, 600 μL of TBARS solution was added to 50 μg of total protein dissolved in 300 μL of Milli-Q water. The mix was incubated for 40 min at 95 °C. Afterward, the sample was centrifuged at 14,000 rpm for 2 min and the supernatant was analysed spectrophotometrically, at 532 nm.

### 2.5. Mitochondrial Trans Membrane Potential by Flow Cytometry and Confocal Microscopy

The mitochondrial transmembrane potential of fresh isolated MNCs was analyzed both by flow cytometry, for evaluating the mean mitochondrial fluorescence intensity of the sample population, and by confocal microscopy for evaluating the intracellular mitochondrial distribution.

For flow cytometric evaluation, the fresh isolated MNCs were washed once with RPMI medium, incubated with 200 nM tetramethylrhodamine methyl ester (TMRM) (Invitrogen, Milan, Italy) for 10 min at 37 °C and immediately measured on a FacsCalibur flow cytometer (Becton Dickinson, San José, CA, USA). To exclude the unspecific staining, the same experiments were conducted in the presence of 50 nM of carbonyl cyanide-4-(trifluoromethoxy)phenylhydrazone (FCCP), an uncoupling molecule. The analysis was confined to viable cells only, after gating procedures based on forward- and side-scatter features. Ten thousand cells per sample were analyzed.

For fluorescence imaging, freshly isolated MNCs were incubated with 100 nM MitoTracker Deep Red-633 (ThermoFisher Scientific Inc.) for 15 min at 37 °C, placed on a coverslip, and immediately analyzed using a Leica TCS SP2-AOBS confocal microscope (Leica Microsystem, Heidelberg, Germany) equipped with a 633 He-Ne laser and Leica specific software.

### 2.6. Proteomic Setup

Samples were solubilized in 40 ul 2% SDC, 40 mM Chloroacetamide, 10 mM TCEP and 100 mM Tris-HCl pH 8 at 95 °C for 10 min and sonicated with a Ultrasonic Processor UP200St (Hielscher), 3 cycles of 30 s. Lysate samples were digested with 0.7 ug Trypsin and 0.3 μg LysC, overnight at 37 °C. Digested samples were processed by in-Stage Tip method using an enclosed Stage Tip, containing 2 poly (styrene-divinylbenzene) reverse-phase sulfonate discs (SDB-RPS) [36].

Elution was performed on an Ultimate 3000 RSLC with an EASY spray column (75 μm × 50 cm, 2 μm particle size, Thermo Scientific) at a flow rate of 250 nL/min with a 180 min non-linear gradient of 6–45% solution B (80% ACN, 20% H2O, 5% DMSO and 0.1% FA). Eluting peptides were analyzed using an OrbitrapVelos Pro mass spectrometer (Thermo Scientific Instruments) in positive ionization mode. Single MS survey scans were performed in the Orbitrap, recording a mass window between 375 and 1500 *m*/*z* using a maximal ion injection time of 50 ms. The resolution was set to 100,000 and the automatic gain control was set to 1,000,000 ions. The experiments were done in data-dependent acquisition mode with alternating MS and MS/MS experiments. A maximum of 10 MS/MS experiments were triggered per MS scan.

Data processing was performed by MaxQuant software version 1.6.1.0 [37]. A false discovery rate was set to 0.01 for the identification of proteins, and a minimum of 6 amino acids was required for peptide identification. The Andromeda engine was used to search MS/MS spectra against Uniprot human database (release UP000005640_9606 August 2017). The intensity values were extracted and statistically evaluated using the ProteinGroup Table and Perseus software version 1.6.1.3 [38]. Algorithm MaxLFQ was chosen for the protein quantification with the activated option “match between runs” to reduce the number of the missing proteins.

The mass spectrometry proteomics data were deposited in the ProteomeXchange Consortium via the PRIDE [39] partner repository with the dataset identifier PXD021960 (reviewer account details: Username: reviewer_pxd021960@ebi.ac.uk; Password: tFCOHhEx).

### 2.7. qPCR Analyses

RNA extraction from MNCs from CCS or healthy donor was performed using the RNeasy Mini Kit (Qiagen, Milan, Italy) and quantified using a NanoDrop spectrophotometer (Nanodrop Technologies, Wilmington, DE, USA). The cDNA was synthesized by using iScript cDNA Synthesis Kit (Bio-Rad Laboratories, Milan, Italy) starting from 1 μg of total RNA. PCR primers were designed through Beacon Designer 2.0 Software (Bio-Rad Laboratories). CLUH primers sequences were TACATCATGGGCGACTACGC (forward primer) and GGCCAGGTGCATGTATTCCT (reverse primer); PGC1-alpha primers sequences were: CTGTGTCACCACCCAAATCCTTAT (forward primer) and TGTTCGAGAAAAGGACCTTGA (reverse primer); Sirt6 human primers sequences were: CCTCCTCCGCTTCCTGGTC (forward primer) and GTCTTACACTTGGCACATTCTTCC (reverse primer). Quantitative real-time PCR (qPCR) was performed in an iQ5 real-time PCR detection system (Bio-Rad Laboratories) using 2× iQ Custom Sybr Green Supermix (Bio-Rad Laboratories). Values were normalized on mRNA expression of human β-actin and HPRT (reference genes). Statistical analysis of the qPCR was performed using the iQ5 Optical System Software version 1.0 (Bio-Rad Laboratories) based on the 2−ΔΔCt method. The dissociation curve for each amplification was analyzed to confirm the absence of nonspecific PCR products.

### 2.8. Western Blot Analysis

Expression of CLUH, PGC-1α, and SIRT6 proteins was determined by Western blot (WB), using standard procedures. MNCs were lysed using lysis buffer (150 mM NaCl, 20 mM TRIS-HCl pH 7.4, 2 mM EDTA, and 1% NP40) containing a protease inhibitor cocktail for mammalian cells (Sigma) and total protein was measured by Bradford assay [40]. After SDS-PAGE, performed according to the standard method on 4–20% polyacrylamide gels, proteins were transferred to a nitrocellulose membrane (BioRad Laboratories). The membrane was blocked for 1 h with PBS-0.1% Tween 20 (PBSt) containing 5% non-fat dry milk and incubated overnight at 4 °C with the following primary antibodies: anti-CLUH (1:1000, cod: A301-764A, Bethyl Lab), anti-PGC-1α (1:1000, cod: ab77210, Abcam), and anti-SIRT6 (1:1000, cod: ab88494, Abcam).

After washing with PBSt, the membrane was incubated with an anti-rabbit or anti-mouse IgG antibody conjugated with horseradish peroxidase (BioRad Laboratories) and developed with Clarity Western ECL Substrate (BioRad Laboratories). Bands were detected and analyzed for density using an enhanced chemiluminescence system (Alliance 6.7 WL 20 M, UVITEC, Cambridge, UK) and UV1D software (UVITEC). Bands of interest were normalized for actin level in the same membrane.

### 2.9. Statistical Analysis

Comparison between CCS samples and healthy age-matched population or elderly healthy subjects was performed via non-parametric statistical approach (Mann–Whitney *U* test). Analyses were performed using the GraphPad Prism version 5.00 statistical software (GraphPad Software Inc., La Jolla, CA, USA). Values of *p* < 0.05 were considered significant.

## 3. Results

### 3.1. CCS-MNCs Showed Altered Glucose Metabolism and Increased Oxidative Stress Production in Comparison to the Age-Matched Control Cells

To investigate the differences in mitochondrial metabolism between CCS and healthy control samples, we have evaluated several biochemical markers in MNCs isolated from peripheral blood.

CCS were stratified into three different groups, based on the age at analysis (Group 1 = <10 y.o., Group 2 = between 11 and 20 y.o., Group 3 = between 21 and 40 y.o.) and compared with age-matched healthy subjects and adult and elderly controls (41–60, 61–80, and >80 y.o.) (Table 1).

The efficiency of oxidative phosphorylation (OxPhos) was evaluated in terms of P/O value, calculated as the ratio between the nmol of produced ATP and the nmol of consumed oxygen. This study was conducted in the presence of pyruvate + malate (Pyr + Mal), or succinate, to stimulate the pathways formed by Complexes I, III, and IV, and Complexes II, III, and IV, respectively.

**Figure 1:**
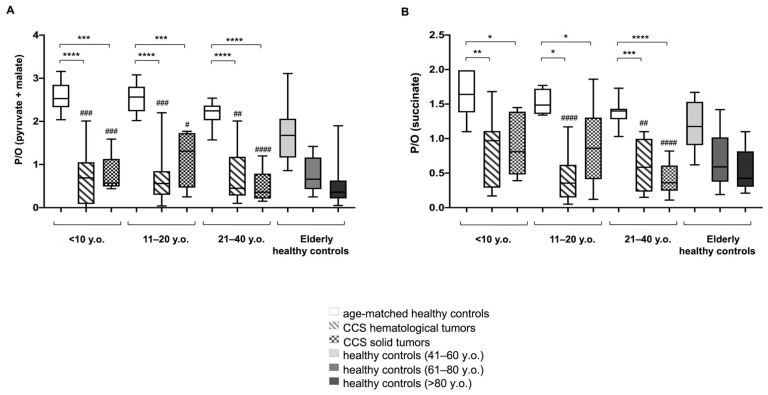
Evaluation of mitochondrial efficiency in MNCs isolated from CCS, and age-matched and elderly healthy controls. (**A**,**B**) reports the P/O value, obtained as the ratio between ATP synthesis and oxygen consumption, in the presence of pyruvate + malate or succinate, respectively, in MNCs isolated from: age-matched healthy controls (<10 y.o. *n* = 17; 11–20 y.o. *n* = 18; 21–40 y.o. *n* = 24), CCS of hematological tumors (<10 y.o. *n* = 26; 11–20 y.o. *n* = 33; 21–40 y.o. *n* = 30), CCS of solid tumors (<10 y.o. *n* = 22; 11–20 y.o. *n* = 29; 21–40 y.o. *n* = 10), adult healthy controls (*n* = 21; 41–60 y.o.), and elderly healthy control (61–80 y.o. *n* = 22 and > 80 y.o. *n* = 25). Statistical analysis was performed via non-parametric statistical approach (Mann–Whitney *U* test). *, **, ***, and **** indicate a *p*-value of *p* < 0.05, 0.01, 0.001, and 0.0001, respectively, between CCS samples and age-matched healthy controls. #, ##, ### and #### indicate a significant difference for *p* < 0.05, 0.01, 0.001, and 0.0001, respectively, between CCS samples and adult healthy controls (41–60 y.o.). No significant differences have been observed between CCS and elderly control (61–80, and >80 y.o.).

As shown in Figure 1A, the P/O ratios after Pyr + Mal stimulation were lower in CCS patients affected by either solid or hematological tumors compared with the respective age-matched controls. In addition, the CCS P/O ratio was lower compared to the healthy population, but it was superimposable with that of subjects of 60 years older. Similar results were obtained when evaluating the P/O ratio after succinate stimulation (Figure 1B).

**Figure 2:**
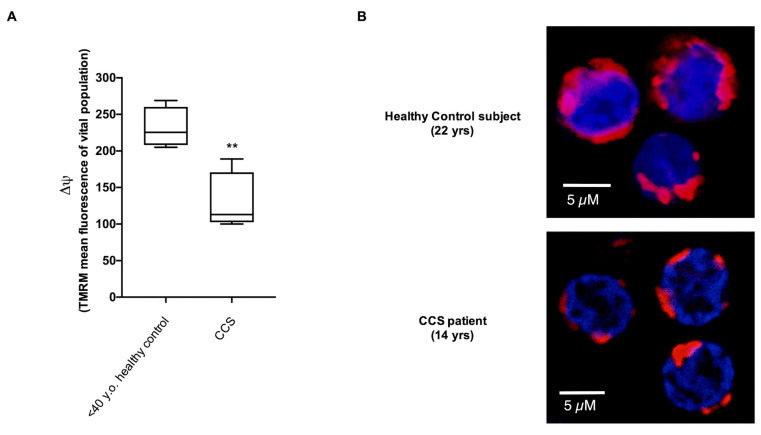
Evaluation of mitochondrial membrane potential in MNCs isolated from CCS and age-matched healthy controls. (**A**) reports data on the mitochondrial transmembrane potential ΔΨ of MNCs isolated from age-matched healthy controls and CCS patients, stained with the fluorescent probe TMRM and analyzed by flow cytometry. Data were derived from five independent experiments. Statistical analysis was performed via a non-parametric statistical approach (Mann–Whitney *U *test), and ** indicates a *p*-value of *p* < 0.01. (**B**) shows a representative confocal microscopy field of MNCs isolated from one healthy subject (22 yrs) and from one CCS patient (14 yrs), stained with Mitotracker deep red 633 (virtual red color) as a marker of the mitochondrial membrane potential, and DAPI (blue color) to highlight the nuclei. Unlike in normal MNC, the mitochondria of CCS samples were distributed as single scattered spots.

These data were confirmed by flow cytometric and confocal microscopy analysis of the mitochondrial membrane potential (MMP), evaluated with specific fluorescent probe TMRM. Data show a decrement of about 44% of MMP levels in CCS with respect to those observed in the healthy age-matched subjects (Figure 2A). The intracellular distribution of mitochondria in CCS cells appeared to be altered as well. Although a pattern of mitochondria is not easily resolved in these cells that display a low cytoplasm/nucleus ratio, we observed a speckled distribution in CCS cells much different from the more uniformly distributed fluorescence in normal MNCs (Figure 2B), suggesting a loss in the mitochondrial network organization.

**Figure 3:**
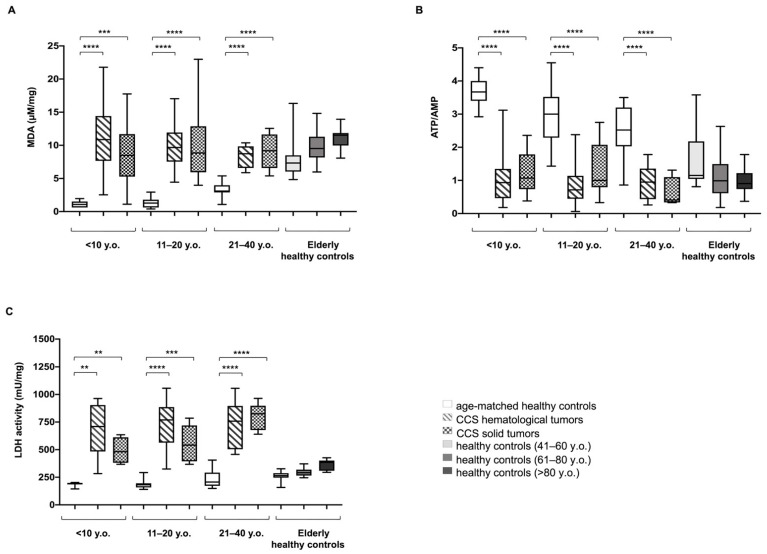
Lipid peroxidation, energy status, and lactate dehydrogenase activity in MNCs isolated from CCS, and age-matched and elderly healthy controls. All data reported in this figure have been obtained using MNCs isolated from: age-matched healthy controls (<10 y.o. *n* = 17; 11–20 y.o. *n* = 18; 21–40 y.o. *n* = 24), CCS of hematological tumors (<10 y.o. *n* = 26; 11–20 y.o. *n* = 33; 21–40 y.o. *n* = 30), CCS of solid tumors (<10 y.o. *n* = 22; 11–20 y.o. *n* = 29; 21–40 y.o. *n* = 10), adult healthy controls (41–60 y.o. *n* = 21;.), and elderly healthy controls (61–80 y.o. *n* = 22 and >80 y.o. *n* = 25). (**A**) shows the cellular level of malondialdehyde (MDA), a marker of lipid peroxidation. Data are expressed as μM/mg of total protein. (**B**) reports the cellular energy status (ATP/AMP), evaluated as the ratio between the intracellular levels of ATP and AMP. (**C**) shows the activity of lactate dehydrogenase (LDH), the marker of lactate fermentation. Data are expressed as mU/mg of total protein. Statistical analysis was performed via non-parametric statistical approach (Mann–Whitney *U* test). **, ***, and **** indicate *p*-values of *p* < 0.01, 0.001, and 0.0001, respectively, between CCS samples and age-matched healthy controls. No significant differences were observed between CCS and adult and elderly controls (41–60, 61–80, and >80 y.o.).

Inefficient mitochondrial metabolism is often associated with an increment in oxidative stress production [41,42,43]. In CCS-MNC, the ROS production was ten-fold higher than in age-matched control cells (30.32 ± 5.39 vs. 3.67 ± 0.90 mean fluorescence, respectively, *p* < 0.0001) (Supplementary Figure S1). Moreover, CCS patients affected either by solid or hematological tumors, displayed levels of malondialdehyde (MDA), a marker of lipid peroxidation [28], higher than age-matched healthy controls, but similar to those reported for adult or elderly healthy controls (Figure 3A).

Since OxPhos is the principal source of ATP production, we have evaluated the energy status of MNCs, analyzing the ATP/AMP ratio (Figure 3B). CCS showed a decreased energy status compared to age-matched healthy controls. This decrease was statistically significant compared to the young age-matched groups, but no significant difference was observed between CCS samples and elderly healthy controls.

We have previously demonstrated that mitochondria lose part of their energetic efficiency during aging, with a corresponding increase of lactate dehydrogenase (LDH), a marker of lactate fermentation, determining a metabolic switch from aerobic to anaerobic energy production [28].

Here, we observed a significant increase in LDH activity in CCS-MNCs from hematological or solid tumor survivors, in subjects of all groups we tested (Figure 3C).

**Figure 4:**
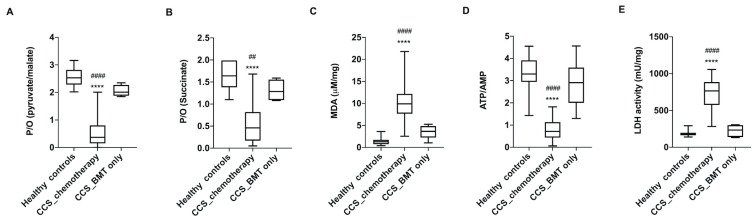
The glucose metabolism alterations were detectable in CCS after chemo/chemoradiotherapy but not after only bone marrow transplantation. The reported biochemical parameters were gathered from the MNCs isolated from hematological CCS patients treated with chemo/chemoradiotherapy (<20 y.o., *n* = 59), CCS patients treated only with bone marrow transplantation (BMT) (<20 y.o., *n* = 15), and the age-matched healthy subjects (*n* = 35). (**A**,**B**) reports the P/O value, obtained as the ratio between ATP synthesis and oxygen consumption, in the presence of pyruvate + malate or succinate, respectively. (**C**) shows the MDA level, as marker of lipid peroxidation. (**D**) reports the cellular energy status, expressed as the ATP/AMP ratio. (**E**) shows the LDH activity, as marker of anaerobic glycolysis. Statistical analysis was performed via non-parametric statistical approach (Mann–Whitney *U* test). **** indicates a *p*-value of *p* < 0.0001 between healthy samples and CCS patients treated with chemo/chemoradiotherapy. ## and #### indicate significant differences for *p* < 0.0001 between CCS patients treated with only BMT and CCS patients treated with chemo/chemoradiotherapy. No significant differences have been observed between healthy subjects and CCS patients treated with only BMT.

The alterations in glucose metabolism were observed only in the CCS patients who underwent chemo/chemoradiotherapy, whereas patients with hematological cancer who underwent allogeneic bone marrow transplantation showed no metabolic changes compared to the healthy subjects of the same age (Figure 4). This was expected, since the transplanted cells from healthy donors did not experience any chemo/chemoradiotherapy. However, when clustering these biochemical parameters based on the cancer types or on the time since the last chemo/chemo-radiotherapy (range: 2 months–25 years), no significant differences were observed among the different analyzed groups (Supplementary Figure S2).

### 3.2. The Metabolic Alterations in CCS Are Suggestive of Premature Aging

**Figure 5:**
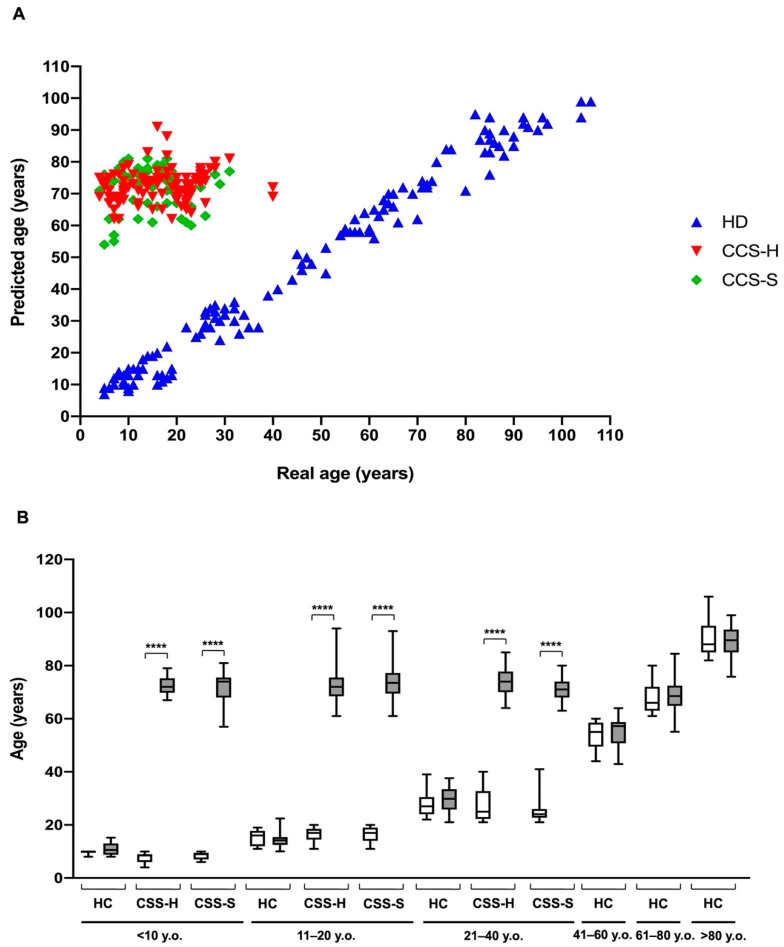
Comparison of real and predicted ages in MNCs isolated from CCS and age-matched and elderly healthy controls. The predicted age was obtained by applying a mathematical model developed by a machine learning method and based on several parameters of glucose metabolism [28]. The data were obtained by evaluating the biochemical parameters of MNCs isolated from: age-matched healthy controls (<10 y.o. *n* = 17; 11–20 y.o. *n* = 18; 21–40 y.o. *n* = 24), CCS of hematological tumors (<10 y.o. *n* = 26; 11–20 y.o. *n* = 33; 21–40 y.o. *n* = 30), CCS of solid tumors (<10 y.o. *n* = 22; 11–20 y.o. *n* = 29; 21–40 y.o. *n* = 10), adult healthy controls (*n* = 21; 41–60 y.o.), and elderly healthy controls (61–80 y.o. *n* = 22 and > 80 y.o. *n* = 25). (**A**) shows the distribution of healthy control (HC, blue), CCS of hematological tumors (CCS-H, red), and CCS of solid tumors (CCS-S, green) in relation to real and predicted age. (**B**) reports the comparison between the real and predicted ages within the same population, subdivided on the basis of decades. Statistical analysis was performed via non-parametric statistical approach (Mann–Whitney *U* test). **** indicates a *p*-value of *p* < 0.0001 between real and predicted age.

Biochemical data suggest that alterations in cellular metabolism, observed in CCS after chemotherapy and radiotherapy, were similar to those observed in elderly subjects. Thus, utilizing our recently developed model to predict age, based on specific glucose metabolism parameters, such as ATP/AMP ratio, P/O ratio in the presence of Pyr + Mal or succinate, MDA level, and LDH activity [28], we have calculated the “ages” of CCS subjects with hematological and solid tumors, age-matched (<40 y.o.), and older healthy controls (>61 y.o.); further, we compared those values to the real ages of these subjects. As shown in Figure 5A, the ages of healthy subjects computed by the mathematical model well correlate with the real ages (Sperman r = 0.98). In contrast, the correlation between predicted and real age was totally lost for CCS samples (Sperman r = 0.15 and 0.07 for hematological and solid tumors, respectively). Notably, the most striking result consists in the observation that the calculated ages for CCS was by far higher than their real ages (Figure 5).

### 3.3. Proteomic Analysis Confirmed Altered Mitochondrial Metabolism

**Figure 6:**
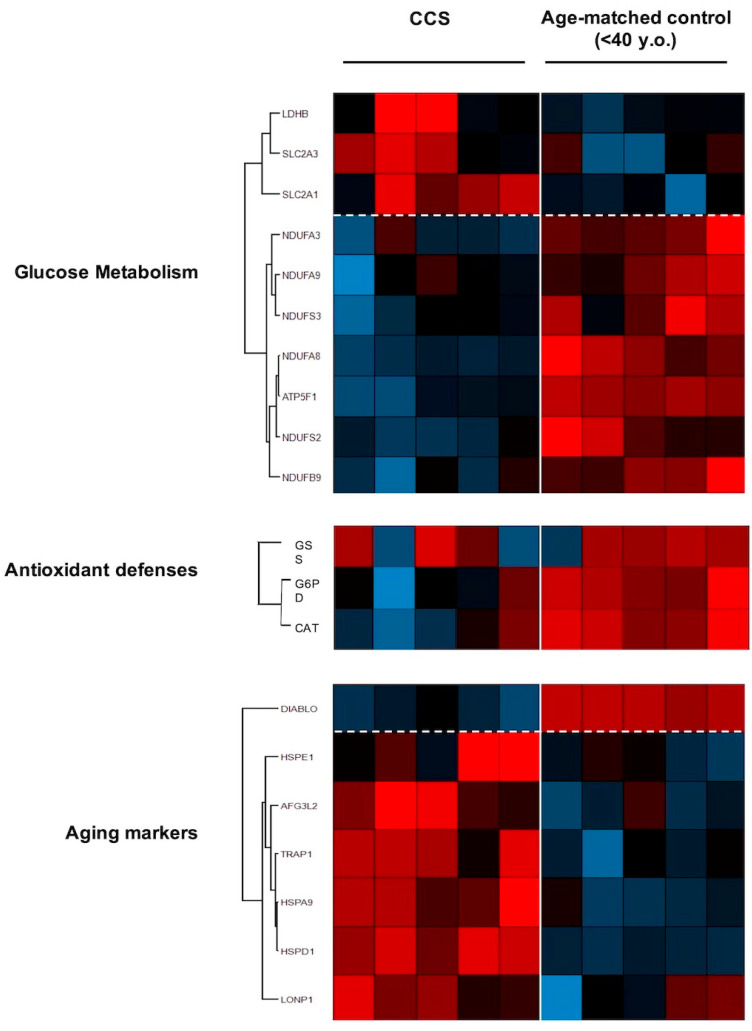
Proteomic analysis of MNCs isolated from CCS and age-matched healthy controls. The figure shows a representative expression heat map of proteins involved in glucose metabolism, antioxidant proteins, and aging markers. Data are expressed as ratios over mean values for the two conditions (red = expression above mean, black = expression at mean; blue = expression below mean). Each datum represents five independent experiments.

The proteomic analysis approach (Figure 6) confirmed that CCS samples were characterized by altered glucose metabolism. In particular, data show higher expression levels in CCS of some proteins involved in the anaerobic metabolism (LDHB) and in the transport of glucose (SLC2A3 and SLC2A1) than in the age-matched controls, confirming the switch from mitochondrial metabolism to anaerobic glycolysis. Reciprocally, the expression of some subunits of respiratory Complex I and ATP synthase and some enzymes involved in the antioxidant defenses, such as glutathione synthase (GSS), catalase (CAT), and glucose 6-phosphate dehydrogenase (G6PD) appeared downregulated in CCS in comparison to the controls.

Finally, we investigated the regulation of expression of proteins involved in the aging processes. We observed downregulation of DIABLO, an apoptosis-inducing protein; and upregulation of proteins involved in stress adaptation (HSPA9), negative modulation of OxPhos (TRAP1), and mitochondrial protein importation, folding, and degradation (i.e., HSPD1, HSPE1, AFG3L2, LONP1), which are suggestive of impaired mitochondrial function.

### 3.4. Genes Involved in Mitochondrial Biogenesis and Activity Regulation Are Altered in CCS

**Figure 7:**
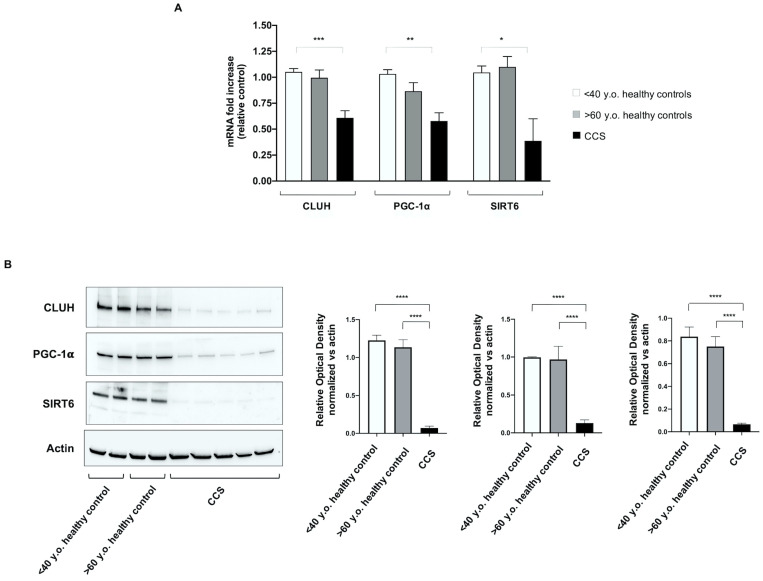
Evaluation of gene and protein expression of CLUH, PGC1-α, and SIRT6 in MNCs isolated from CCS and age-matched and elderly healthy controls. In (**A**) the graph reports the genomic expression of CLUH, PGC1-α, and SIRT6 in MNCs isolated from age-matched healthy controls (<40 y.o. *n* = 10), CCS (<40 y.o. *n* = 16), and elderly healthy controls (>60 y.o. *n* = 12). (**B**) reports the WB signals against CLUH, PGC1-α, and SIRT6 (left) and the relative densitometric analyses (right) of MNCs isolated from age-matched healthy controls (<40 y.o.), elderly healthy controls (>60 y.o.), and CCS (<40 y.o.). Data are representative of four independent experiments each and are expressed as mean ± SD. The values of WB signals were normalized against an actin signal. Statistical analysis was performed via non-parametric statistical approach (Mann–Whitney *U* test). *, **, *** and **** indicate *p*-values of *p* < 0.05, 0.01, 0.001, and 0.0001, respectively, between CCS samples and age-matched healthy controls. No significant differences were observed between age-matched and elderly healthy controls. Original blots see Supplementary Figure S3.

To disclose possible mechanisms involved in the metabolic alterations observed in CCS, the expression of several genes involved in mitochondrial function has been investigated. Our panel included genes involved in (i) mitophagy (PINK1, PARKIN), (ii) mitochondrial fusion/fission processes (MNF1 and FIS1), (iii) mitochondrial biogenesis (CLUH and NRF1), and (iv) regulation of mitochondrial metabolic pathways (PGC1-α, SIRT1, and SIRT6). CCS showed significant downregulation of CLUH, PGC1-α, and SIRT6 gene expression, in comparison to both control groups (age-matched and elderly) (Figure 7A). In other words, altered expression of these genes appears to be a peculiar sign of CCS, not linked with the “physiological aging.” Conversely, the gene expression of PINK1, PARKIN, MNF1, FIS1, NRF1, and SIRT1 appeared uncompromised in CCS and similar to healthy control samples.

The qPCR results were confirmed by the WB analysis, which shows that the protein expression levels of CLUH, PGC1-α, and SIRT6in cell lysates were similar in young and elderly healthy subjects, but were hardly detectable in CCS samples (Figure 7B).

## 4. Discussion

Progress in the treatment of children with cancer has led to a remarkable increase in the number of survivors living into adulthood. Despite this impressive success, the impacts on the long-term health of these individuals remain substantial, with most experiencing chronic health conditions related to previous treatment [44]. Late effects of chemo/chemoradiotherapy [45,46,47] are reflected in outcomes typically associated with aging, such as minimal cognitive dysfunction, reduced muscle strength, cardiovascular disease, poor exercise tolerance, increased cellular senescence, reduced telomere length, epigenetic modifications, somatic mutations, and mitochondrial damage [8,9,10,11,12,13,14].

Therefore, the objective of the present work was to identify molecular and biochemical alterations to blood cells in order to give a pathophysiological basis for frailty symptoms observed in CCS. Our attention focused on the mitochondrial energy metabolism and the relative oxidative stress production, since the reactive oxygen species is one of the principal causes of aging [48].

Our data show that CCS-MNCs are characterized by an impairment of OxPhos efficiency, evaluated in term of P/O ratio ad MMP, due to the uncoupled status between the oxygen consumption and ATP synthesis, in comparison to the age-matched controls; indeed, the P/O ratios were very similar to those observed in the elderly control (>60 y.o.). This aerobic metabolism inefficiency was observed to be associated with (i) lower expression levels of some subunits of Complex I (NDUFA3, NDUFA9, NDUFS3, NDUFA8, NDUFS2, and NDUFB9) and ATP synthase (ATP5F1); (ii) over-expression of several heath-shock proteins (HSP) involved in the mitochondrial protein importation, folding, and degradation (i.e., HSPE1 [49], HSPD1 [50], AFG3L2 [51], and LONP1 [52]), and in stress adaptation (i.e., HSPA9 [53]).

The metabolic switch observed in our analysis was further confirmed by the increased expression of TRAP1, a negative modulator of OxPhos metabolism, and an inducer of glycolysis [54]. By contrast, the expression of DIABLO, a protein favoring the cellular apoptosis [55], appeared lower with respect to the age-matched control cells, confirming that programmed cell death is impaired in senescent cells [56]. Thus, the accelerated frailty symptoms in CCS could be associated with the cell senescence [57], since the accumulation of senescent cells for prolonged periods [57] favors the onset of age-related diseases because of their low, but chronic, senescence-associated secretory phenotype [57,58,59].

The CCS-MNCs’ metabolic alteration determines the reduction of chemical energy synthesis influencing the ATP/AMP ratio. Moreover, mitochondrial inefficiency increases oxidative stress production, as confirmed by the high ROS level and the evident accumulation of MDA, a final product of lipid peroxidation, suggesting that the cellular antioxidant defenses are not sufficient to counteract the increased oxidative stress. This hypothesis was further corroborated by the proteomic analysis that showed lower expression levels of G6PD, GSS, and CAT, enzymes involved in the antioxidant response. Moreover, considering that the integrity of the inner mitochondrial membrane is essential for efficient OxPhos and that mitochondria are major sources of oxidative stress, the decrement of antioxidant defenses associated with the localized oxidative stress production could cause structural damage in the inner mitochondrial membrane, exacerbating the ROS production. In turn, this could trigger a vicious circle in which ROS production becomes both cause and effect of mitochondrial dysfunction [60]. Of note, the OxPhos inefficiency is more evident in the presence of pyruvate/malate than with the induction by succinate. This could be explained considering that pyruvate + malate activates the pathway triggered by Complex I, which is the principal source of ROS production [61,62]. Conversely, succinate, stimulating Complex II, is less involved in oxidative stress production.

In response to mitochondrial dysfunction, CCS-MNCs increase the expression and activity of LDH and the expression of the glucose carriers (SLC2A3 and SLC2A1), to compensate the altered energy balance. This switch from aerobic to anaerobic metabolism is imposed by two cellular needs: (i) the restoration, at least in part, of the ATP levels, by incrementing the glucose catabolism; (ii) the balancing of the NADH/NAD^+^ ratio. The recycling of NADH to NAD^+^ is essential to avoid glycolysis slow-down [63], and reductive stress accumulation, which could increment oxidative stress [64]. In fact, in healthy cells, the correct NADH/NAD^+^ ratio is mainly maintained by Complex I activity, but in the case of mitochondrial dysfunction, this task is performed exclusively by LDH [63].

To further extend the investigation on the possible causes of the metabolic mitochondria alteration, a panel of genes involved in mitochondrial biogenesis, function, and regulation were evaluated. Results showed that the genes involved in mitophagy, fusion, and fission processes (PINK1, PARKIN, MFN1, FIS1, and NRF1) were apparently unimpaired with respect to the controls, whereas genes involved in mitochondrial function, such as CLUH, PCG1-α, and SIRT6, were remarkably less expressed. These data were confirmed by the analysis of protein expression. SIRT6 and PGC1-α play pivotal roles in the regulation of energy metabolism: SIRT6 determines a low level of HIF-α, favoring aerobic metabolism [65]; PGC1-α modulates both the biogenesis and the composition and functions of individual mitochondria [66]. CLUH is a gene encoding a cytosolic RNA-binding protein involved in the distribution of mitochondria inside the cell and linked to the efficiency of mitochondria OxPhos [32,67]. Therefore, the CLUH low expression is in line with the poor OxPhos activity observed by biochemical analysis. Moreover, these results corroborate previous observations on the association between CLUH and mitochondria function [32,67,68]. Interestingly, the alterations of CLUH, SIRT6, and PGC1-α were not observed in elderly controls, despite the metabolic impairment, suggesting that the biochemical mechanism of aging runs in different ways, at least in some relevant aspects in CCS compared to the aging as a “physiological process.”

The early aging of CCS was confirmed by the application of our mathematical model based on glucose metabolism and oxidative stress parameters [28]. In particular, results showed that the predicted ages were very different in comparison to the real ages for CCS patients treated both for hematological and solid tumors. In contrast, by utilizing our mathematical model, the predicted ages of healthy controls were not much different from the real ages.

To make a general statement about the data reported herein, metabolic dysfunctions and genetic alterations appear to be irrespective of the original cancer type, and of the time elapsed between the last therapy and the time of biochemical and genetic analysis. However, possible relationships between specific types of therapy and MNC biochemical/molecular lesions could not be analyzed because of the low sample sizes of the various therapy subgroups. A clear understanding of the roles of the different therapies in the altered cell metabolic features will require investigation with additional CCS. However, it has to be mentioned that the main focus of the efforts made in the present study was the elucidation of metabolic and genetic features of MNCs of overall CCS subjects. Moreover, our data seem to suggest no recovery of the biochemical injury with time, suggesting possible permanent damage in hematopoietic stem cells (HSC). Unfortunately, because the riddle of whether HSC are all active at the same time has not yet been solved [69,70], it is very difficult to address this topic.

Another aspect to be taken into account is that the lifespan and the mitochondrial density of MNCs are different in comparison to those of other organs, such as liver and heart [71], although the analysis of MNCs represents a good model for evaluating the health status of the entire organism [29]. This could imply that other tissues could accumulate different degrees of damage from alterations of mitochondrial metabolism. It is possible that other tissues may harbor biochemical/molecular alterations of different severities, depending on the combination of tissue specificity with the type of therapy and the time elapsed between it and the analysis of biochemical parameters. However, in this work, we have not evaluated cells of tissues other than MNCs, which remains, by far, the most accessible cell source.

A general better response to chemotherapy and higher treatment-related toxicity are both observed in childhood cancer patients than in adults. This could be, at least in part, ascribed to higher expression of the apoptotic protein machinery in infant tissues than in adulthood [72]. Nevertheless, this holds true for liver, brain, heart and kidney tissues, but not for MNCs [72]. The latter display indeed high mitochondrial priming for apoptotic death in adults as well. Accordingly, we may hypothesize that extending this study to the adult cancer survivor counterparts might likely disclose long-term treatment-related mitochondrial dysfunction of MNC.

Finally, we are aware that other molecular alterations may be involved in the early fragility of CCSs in addition to alterations in energy metabolism and subsequent oxidative stress. Specifically, chronic inflammation could be sustained by high level of C-reactive protein, and the imbalance between pro- and antioxidant interleukins and cytokines [73,74]. Moreover, differences in gut microbial composition were found between CCS and their siblings [74]. Specifically, the protective *Faecalibacterium* is depleted in ALL-CCS, predisposing them to obesity and other chronic pathologies [75].

## 5. Conclusions

Our data suggest that one of the causes of the frailty symptoms in CCS could be alterations in aerobic mitochondrial metabolism, related to a defect in the organization of their biogenesis and metabolism modulation. Moreover, the evaluation of glucose metabolism may represent a new no-invasive tool to detect precociously the symptoms predicting aging of CCS related to chemo/radiotherapy. These initial findings warrant further research aimed at discovering additional molecular/biochemical alterations, to distinguish more severe from less severe conditions at the cellular level and possibly correlate them with clinical symptoms.

Finally, this study may facilitate identifying therapies to restore the mitochondrial function, to slow down the onset of frailty symptoms and the associated pathological conditions in CCS.

## Figures and Tables

**Table 1:** Characteristics of CCS and control subjects.

Childhood Cancer Survivors						
Age	Solid Tumors			Haematological Malignancies		
Years	Subjects N°	Female/Male	End of therapy (years)	Subjects N°	Female/Male	End of therapy (years)
<10 (Group 1)	35	19/16	6 (1–9)	32	25/7	2 (1–6)
11–20 (Group 2)	48	18/30	10 (3–17)	38	16/22	4 (1–15)
21–40 (Group 3)	10	2/8	18 (4–25)	33	21/12	9 (2–26)
**Total number**	**93**			**103**		
**Controls**						
**Age-Matched**				**Aged Controls**		
Years	Subjects N°		Female/Male	Years	Subjects N°	Female/Male
<10	29		14/15	41–60	27	22/5
11–20	25		15/10	61–80	27	10/17
21–40	25		15/10	>80	25	17/8
**Total number**	**79**				**75**	

## Data Availability

Data supporting the findings of this study are available from the corresponding author upon reasonable request.

## Supplementary Materials

The following are available online at https://www.mdpi.com/article/10.3390/cancers13205214/s1, Figure S1: Comparison of glucose metabolism alterations in different types of tumors, Figure S2: Comparison of glucose metabolism alterations in different types of tumors, Figure S3. Original blots.
