# Supplementary material for: Identification of Biochemical and Molecular Markers of Early Aging in Childhood Cancer Survivors
Source: Cancers (Basel). 2021 Oct 18;13(20):5214. doi: 10.3390/cancers13205214 (PMC8534026; doi:10.3390/cancers13205214)
Supplement: Supplementary file 1 [file cancers-13-05214-s001.zip › cancers-1402733-supplementary Figure S1,S2.pdf]

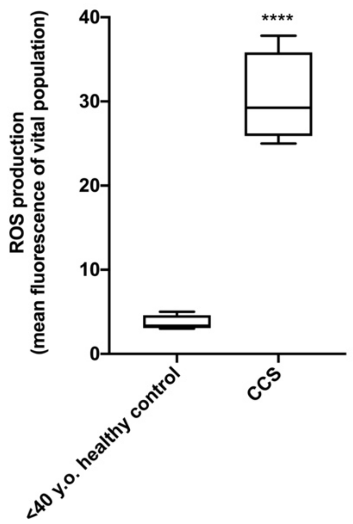

Figure S1. Evaluation of ROS production in MNC isolated from CCS and age-matched healthy control. Graph reports the ROS production in MNC isolated from age-matched healthy controls and CCS patients, stained with the fluorescent probe H2DCFA and analyzed by flow cytometry. Data derive from five independent experiments. Statistical analysis was performed by non-parametric statistical approach (Mann-Whitney U test), and **** indicates a significant difference for $p < 0.0001$.

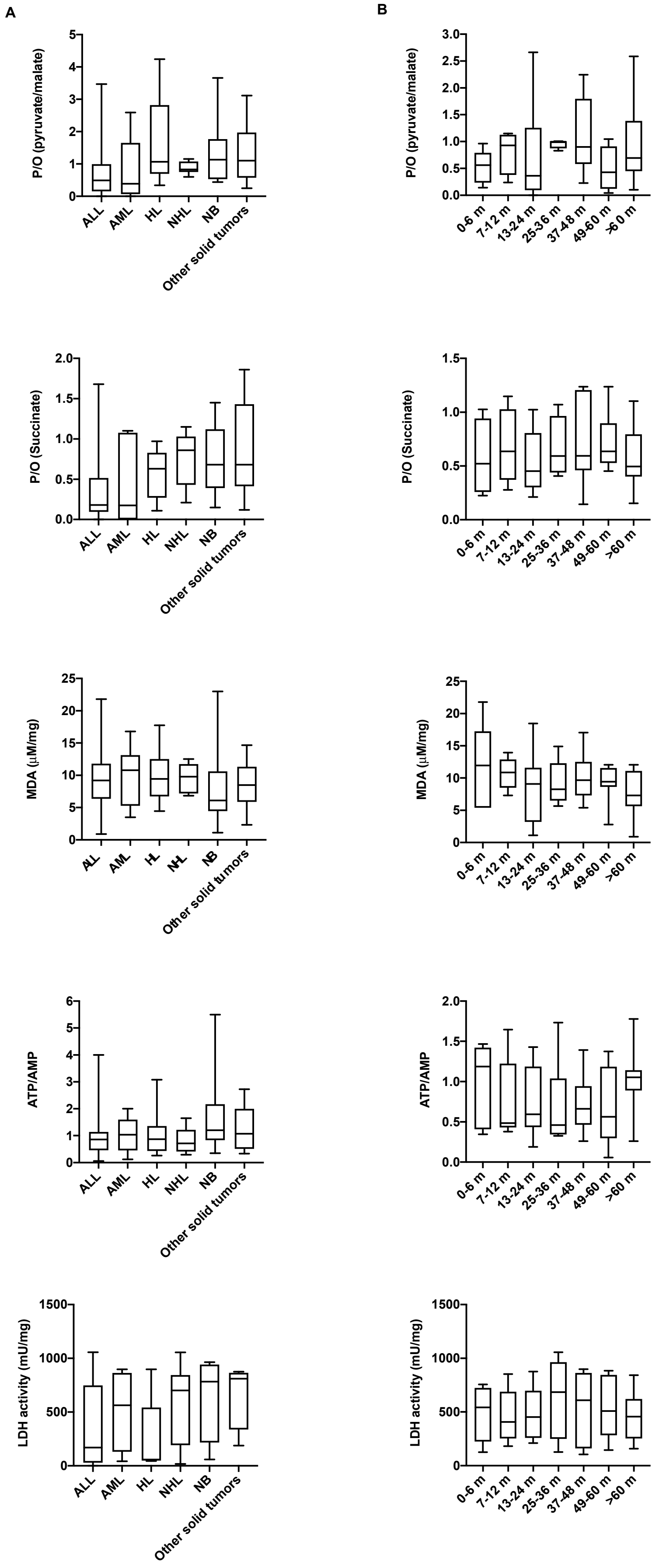

Figure S2. Comparison of glucose metabolism alteration in different type of tumors. Panel **A** shows the P/O values (obtained as the ratio between ATP synthesis and oxygen consumption, in the presence of pyruvate + malate or succinate), MDA concentration, ATP/AMP ratio and LDH activity in MNCs

isolated from patients who survived the following types of tumors: acute lymphocytic leukemia (ALL; n = 42), Acute myeloid leukemia (AML; n = 15), Hodgkin lymphoma (HL; n = 19), Non-Hodgkin lymphoma (NHL; n = 13), Neuroblastoma (NB; n = 34), and other solid tumors (n = 21). Panel **B** reports the same biochemical parameters reported in Panel A in the MNCs isolated from CCS patients clustered based on the time elapsed since the last clinical treatment at the time of blood collection. The time is indicated in months: 0-6 months (n = 12), 7–12 months (n = 20), 13–24 months (n = 15), 25–36 months (n = 34), 37–48 months (n = 24), 49–60 months (n = 18), >60 months (n = 21). Statistical analysis was performed by non-parametric statistical approach (Mann-Whitney U test). No significant differences have been observed among the analyzed samples.
