# Supplementary figures and images for: Identification of Biochemical and Molecular Markers of Early Aging in Childhood Cancer Survivors
Source: Cancers (Basel). 2021 Oct 18;13(20):5214. doi: 10.3390/cancers13205214 (PMC8534026; doi:10.3390/cancers13205214)

## MATERIAL FOR REVIEWER

### Original Western Blot Signal

#### 1) CLUH

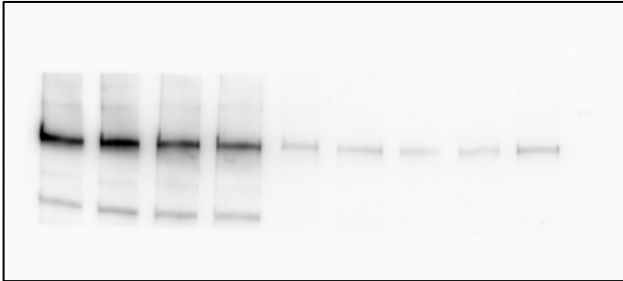

#### 2) PGC-1 $\alpha$

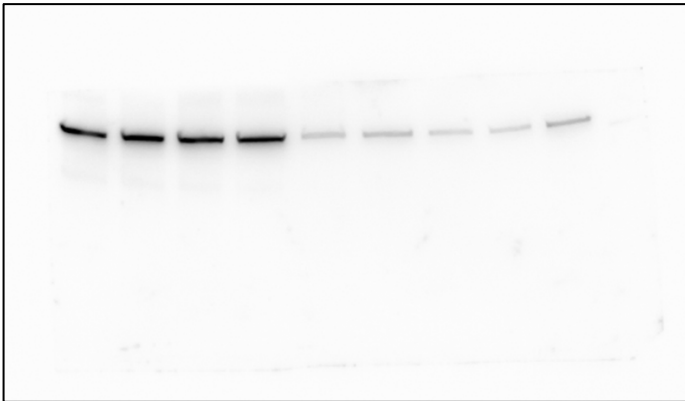

#### 3) SIRT6

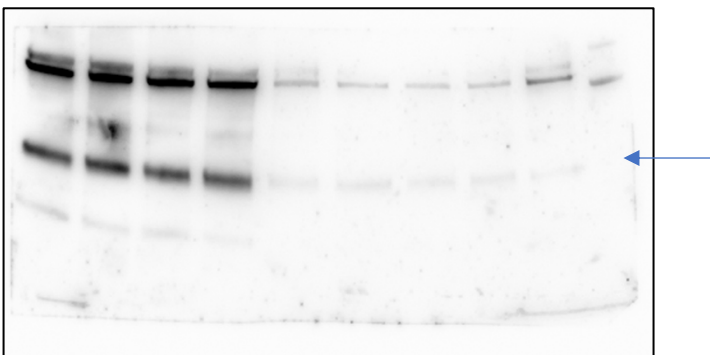

#### 4) Actin

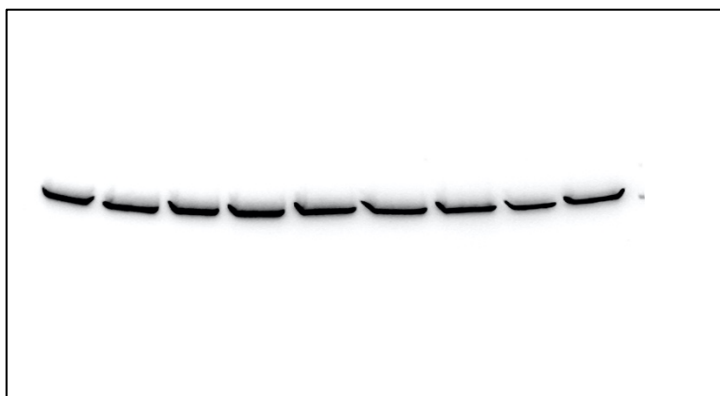

Supplement: Supplementary file 1 [file cancers-13-05214-s001.zip › cancers-1402733-Figure S3-original-images.pdf]
